# Arterial blood gas analysis in dogs with bronchomalacia

**DOI:** 10.1371/journal.pone.0227194

**Published:** 2019-12-31

**Authors:** Yohei Hara, Kenji Teshima, Yoshiki Yamaya

**Affiliations:** Veterinary Anesthesiology & Respiratory Research Laboratory, Department of Veterinary Medicine, College of Bioresource Sciences, Nihon University, Fujisawa, Kanagawa Japan; University of Bari, ITALY

## Abstract

Canine bronchomalacia (CBM) is a structural airway disease leading to chronic cough and intermittent respiratory distress, primarily affecting elderly dogs of small breeds. Results of blood gas analysis have been reported in dogs with several diseases, but not yet in those with CBM. Eleven dogs with CBM were recruited in this study. Most dogs presented with mild hypoxemia and normocapnia, and all with increased alveolar-arterial difference for O_2_ (A-aDO_2_). In computed tomography, abnormal lung patterns, such as atelectasis and parenchymal band, were detected in all dogs, consistent with the regions affected by CBM. We conclude that CBM causes abnormal lung patterns and results in impaired oxygenation. Blood gas analysis is a useful tool for detecting mild pulmonary lesions and concurrent CBM.

## Introduction

Tracheobronchomalacia (TBM) is characterized by airway collapse due to weakness of both tracheal and bronchial walls, supported by their cartilage. Human TBM is classified as either congenital or acquired. Congenital TBM is a common disorder in infants, caused by impaired cartilage maturation or cartilage deficiency [1]. Acquired TBM is caused by airway infection, chronic airway inflammation, endobronchial intubation, or airway compression [2]. The clinical symptoms of both congenital and acquired TBM include cough, dyspnea, and recurrent pneumonia, due to the inability to clear secretion [3, 4]. In veterinary medicine, TBM is also observed, but is usually classified as a complex disease of classical tracheal collapse and bronchomalacia (BM) of the main stem bronchus [5].

Canine BM (CBM) is commonly observed in Poodles, Yorkshire Terriers, and brachycephalic dogs, with the clinical signs consisting of dry cough, cyanosis, and respiratory distress [6, 7]. It mostly affects elderly dogs, and is associated with obesity and cardiomegaly. Thus, CBM may be etiologically different from human acquired TBM. The association between inflammation and CBM is controversial [7]. CBM is also categorized as static or dynamic [5], according to whether the reduction in luminal diameter is constant or changes in luminal diameter are observed during respiration [5]. Radiographic lung abnormalities have been sometimes reported [5, 6, 7, 8], but no profiling of pulmonary function, such as ventilation and oxygenation, has been discussed in the literature.

Blood gas analysis is useful to assess respiratory ventilation, oxygenation, and acid-base balance, thus clarifying the physiological state of the patient [9]. Blood gas analysis has been reported to be useful in assessing the severity of pulmonary diseases, such as pneumonia and pulmonary fibrosis, and of brachycephalic syndrome in dogs [10, 11]. However, it is not known how CBM affects blood gas analysis in dogs. The purpose of this study, therefore, was to assess the degree of pulmonary gas exchange abnormalities in CBM.

## Materials and methods

### Animals

Medical records of dogs with CBM, diagnosed with dynamic computed tomography at the Animal Medical Center of Nihon University between February 2017 and February 2018, were reviewed retrospectively [12].

### Ethics statement

All examinations were performed after written informed consent of the dogs’ owners under the guideline for the care and use of laboratory animals by The College of Bioresource Sciences, Nihon University. In addition, the samples were collected for routine clinical diagnostic purposes by well-educated and trained veterinarian in Animal Medical Center, Nihon University. All dogs were also diagnosed and treated by these veterinarians at the hospital and provided with customary high standards of medical care and welfare. In addition, the study was performed in accordance with the Guide for Animal Experimentation published by the College of Bioresource Sciences, Nihon University for exemption from the requirement for ethics approval.

### Arterial blood gas analysis

One milliliter of arterial blood sampling was performed with the dogs kept as calm as possible. All samples were obtained from the femoral artery with a 25G needle attached to a specific blood gas syringe (PICO70®, radiometer). Samples were acquired in < 1 minute. Arterial blood was analyzed by blood gas analyzer (RAPIDPoint 500®, Siemens Healthcare) immediately after sampling, and the results were corrected for rectal temperature. PaO_2_ values were classified as normoxemia (PaO_2_ ≥ 80 mmHg), mild hypoxemia (60 ≤ PaO_2_ < 80 mmHg), moderate hypoxemia (45 ≤ PaO_2_ < 60 mmHg), or severe hypoxemia (PaO_2_ < 45 mmHg). PaCO_2_ values were classified as normocapnia (32 < PaCO_2_ < 43 mmHg), hypocapnia (PaCO_2_ ≤ 32 mmHg), or hypercapnia (PaCO_2_ ≥ 43 mmHg). A-aDO_2_ values greater than 21 mmHg were considered elevated.

### Dynamic computed tomography

Dynamic computed tomography (CT) of the lung was performed in dogs that had been sedated using medetomidine (Dorbene®, Kyoritsu Seiyaku co. Ltd) 2.5 μg/kg and butorphanol (Vetorphal®, Meiji Seika Pharma co. Ltd) 0.4 mg/kg, administered intramuscularly [13]. Twenty minutes after the injection, the dogs underwent dynamic CT using a 320-slice CT scanner (Aquilion One®, Canon Medical), and CT images were obtained during both the inspiratory and expiratory phases. The dogs were kept in sternal recumbency using a positioning device to straighten the body and the scan was centered at the tracheal bifurcation, with the following parameters: 120 V, 14 mAs, scan time 3s, rotation time 0.275 s, coverage 16 cm. In addition, bronchial diameter during the inspiratory and expiratory phases was measured and compared using the CT imaging viewer. In particular, the short and long axis diameters of the lobar bronchi were measured adjacently to the lobar branches. To avoid oblique cut distortion, the images were adjusted to orthogonally intersect the bronchial axis using multi-planar reconstruction (Aquilion One®, Canon Medical). Briefly, CBM was diagnosed when the height to width ratio of the lobar bronchi was > 2 [12]. Lung patterns were categorized to atelectasis (increased attenuation with the loss of lung volume), consolidation (increased attenuation without the loss of lung volume), grand glass opacity (hazy increased attenuation), mosaic pattern (inhomogeneous lung opacity by areas of relatively increased attenuation and areas of relatively decreased attenuation), emphysema (low attenuation) and parenchymal band (nontapering linear or reticular opacity) based on the criteria of Roels et al and Scrivani et al [14, 15].

## Results and discussion

### Animals

Eleven dogs were included. The breeds included Pugs (n = 3), French Bulldogs (n = 2), Chihuahua, Toy Poodle, Pomeranian, Maltese, Miniature Pinscher, and mixed breed. The median age at presentation was 11 years (range 7–15), and the dogs included 6 neutered males, 4 spayed females, and 1 intact male. The median body weight was 5.4 kg (range 2.6–10.3). Cough was the primary complaint (n = 9), other complaints being persistent respiratory difficulty (n = 5), intermittent respiratory difficulty, gagging, and retching. The median rectal temperatures, heart rates (HR), and respiratory rates (RR) were, respectively, 38.5°C (range 37.9–38.9), 120 beats/min (range 92–180), and 38 breaths/min (range 20–60, excluding three cases with panting). Three dogs had an elongated soft palate. Two dogs had myxomatous mitral valve disease and one of them had cardiogenic pulmonary edema. Two dogs had suspected interstitial pneumonia. Details are shown in S1 Table.

### Arterial blood gas analysis

Arterial blood was collected from all the dogs, and the related findings are presented in Table 1. Eight dogs (73%) were classified as normocapnia, 2 (18%) as hypocapnia, and one (9%) as hypercapnia. Two dogs (18%) were classified as normoxemia, 6 (55%) as mild hypoxemia, 2 (18%) as moderate hypoxemia, and one (9%) as severe hypoxemia. The calculated A-aDO2 was elevated in all dogs.

**Table 1:** Arterial blood gas measurements in CBM (n = 11).

pHa	PaCO_2_ (mmHg)	PaO_2_ (mmHg)	A-aDO_2_ (mmHg)
7.388 (7.297–7.484)	35.8 (23.8–43.7)	63.5 (27.8–92.7)	40.4 (27.6–69.6)

Arterial blood was collected at fraction of inspired oxygen equal to 21%. Data are shown as median (range). pHa, arterial pH; PaCO_2_, carbon dioxide arterial partial pressure; PaO_2_, oxygen arterial partial pressure; A-aDO_2_, alveolar-arterial oxygen difference. Details are shown in S2 Table.

### Computed tomography findings

**Fig 1:**
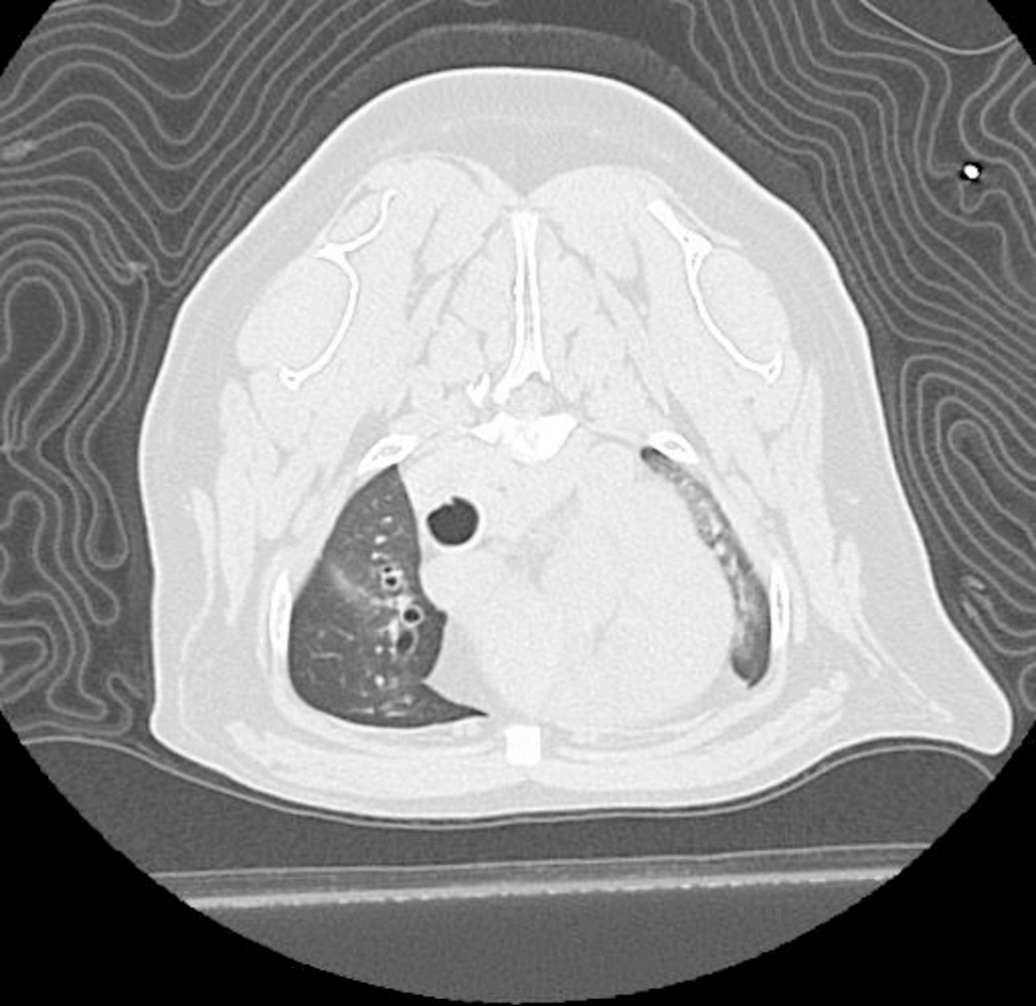
Computed tomography images of case 7 during the inspiratory phase. Atelectasis of the left cranial lobe can be appreciated.

**Fig 2:**
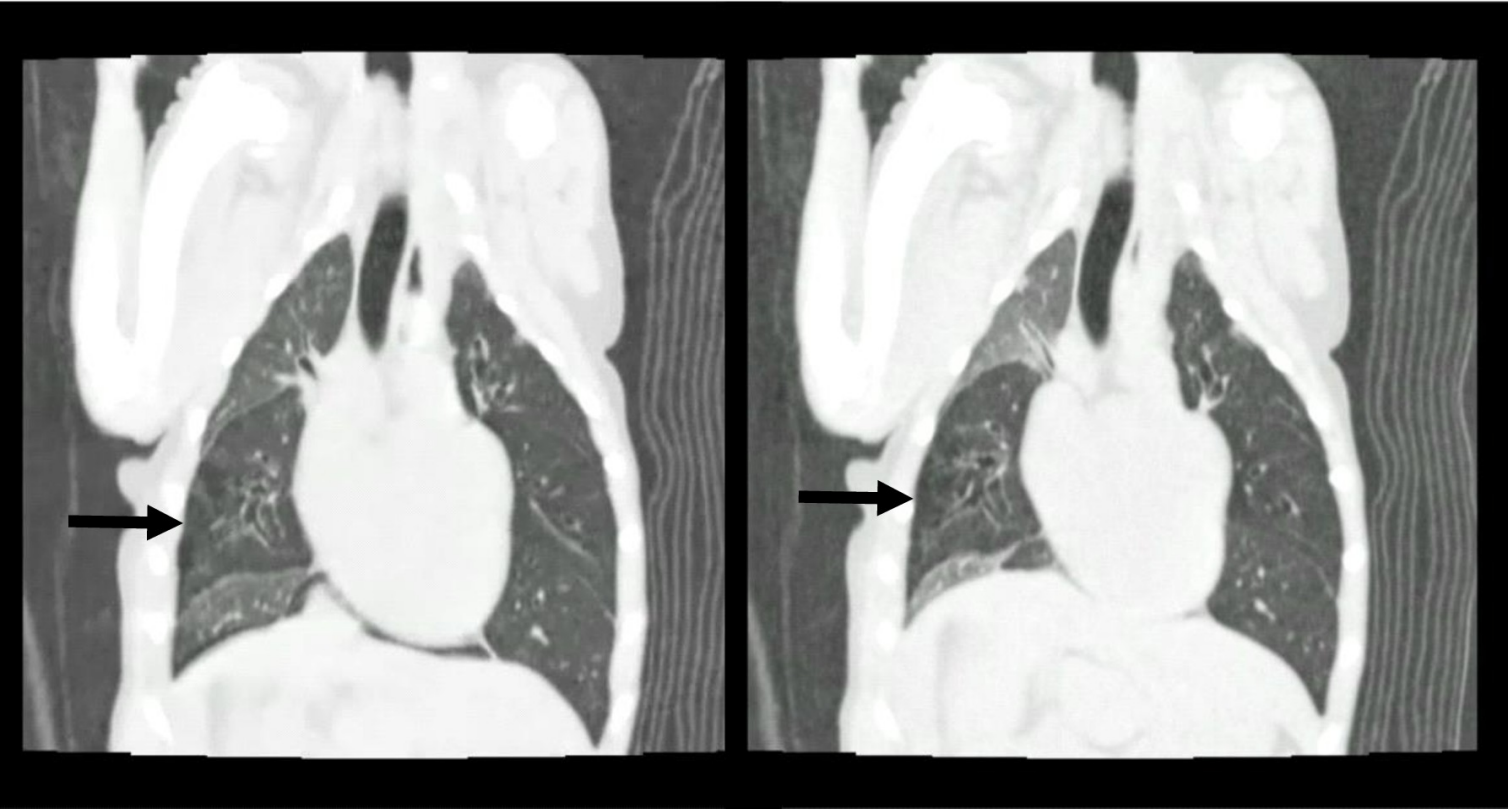
Inspiratory (left) and expiratory (right) images of case 3. The lungs appear hyperlucent except for the right cranial and right caudal lobes. The right middle lobe is affected by pulmonary emphysema (black arrow).

Nine bronchi showed static CBM and 30 dynamic CBM. The bronchus most often affected by CBM was the left cranial lobe bronchus, followed by the right cranial lobe bronchus. Parenchymal band was the most common abnormal lung pattern, followed by atelectasis (Figs 1 and 2). All lung lobes with atelectasis had a bronchus affected by CBM. The findings combined with pulmonary function are presented in Table 2.

**Table 2:** Pulmonary function and dynamic computed tomography findings.

Case	Oxygenation/ Ventilation status	Sites of airway narrowing at inspiratory phase	Sites of airway narrowing at expiratory phase	Lung patterns and area
**1**	Hypocapnia	Carina	Carina, Rcr, Lcr	Consolidation and parenchymal band in Rcr and Lcr.
**2**	Normal	Carina	Carina, Rcr, Rcau, Lcr	Parenchymal band in Rcr and Lcr. Atelectasis in Rcr and Lcr
**3**	Mild hypoxemia	Rmid, Lcr	Rcr, Rmid, Rcau, Acc, Lcr, Lcau	Mosaic pattern during the expiratory phase. Emphysema in Rmid
**4**	Mild hypoxemia	Lcr	Carina, Rmid, Rcau, Acc, Lcr	Parenchymal band in Lcr. Atelectasis in Lcr.
**5**	Mild hypoxemia / Hypercapnia	Acc	Carina, Rcr, Acc, Lcau	Parenchymal band in Rcr.
**6**	Mild hypoxemia	Carina	Carina, Rcr, Rcau, Lcr	Parenchymal band in Rcr and Lcr. Atelectasis in Rcr and Lcr.
**7**	Mild hypoxemia	Lcr, Lcau	-	Atelectasis in Lcr.
**8**	Mild hypoxemia / Hypocapnia	-	Lcr	Atelectasis in Lcr.
**9**	Moderate hypoxemia	-	Rcr, Rmid, Lcr, Lcau	Diffuse GGO.
**10**	Moderate hypoxemia	Lcr, Rcr	Lcr, Rcr	Diffuse GGO.
**11**	Severe hypoxemia	Lcr	Lcr, Lcau	Diffuse GGO. Consolidation in Lcau.

Rcr, Right cranial lobe; Rmid, Right middle lobe; Rcau, Right caudal lobe; Acc, Accessory lobe; Lcr, Left cranial lobe; Lmid, Left middle lobe; Lcau, Left caudal lobe; GGO, Grand glass opacity.

## Discussion

Previous population studies reported that brachycephalic and small breeds, and especially Poodles and Yorkshire Terriers, are the breeds most affected by CBM [5, 6, 7]. Similarly, the present study shows that brachycephalic breeds, such as Pugs and English Bulldogs, were the most commonly affected, followed by small breeds. The body weight distribution reflects the higher prevalence of small breeds. Body condition scores (BCS) were available for 5 of the dogs in the study, and the median BCS was 4/5. It has been reported that obesity in dogs increases airway resistance and reduces the functional residual capacity [16]. We speculate that most of the dogs in this study were obese.

Visualization of airway collapse by bronchoscopy is considered the gold standard for the definitive diagnosis of CBM, but requires sedation or anesthesia and is limited to peripheral small airways or airways beyond a stenosis of the whole lung [17]. Therefore, new tests able to assess both the whole lung and small airways are needed. Recently, dynamic expiration computed tomography has been proposed for the diagnosis of CBM, and can simultaneously detect abnormalities of the whole lung [18]. In veterinary medicine, normal CT can detect bronchial collapse, but the correct respiratory phase cannot be controlled during spontaneous breathing [12]. Compared with standard radiography, fluoroscopy is a dynamic imaging technique, and thus superior in detecting airway collapse [19]. Thus, sequential images are important in diagnosing CBM. Dynamic CT can distinguish the respiratory phases thanks to the continuous scanning and detect both static and dynamic CBM. In children, dynamic CT has excellent sensitivity and great specificity in detecting TBM [18].

Six out of the nine dogs affected by dynamic CBM had tracheal collapse at the tracheal bifurcation. A previous study suggested that dynamic CBM is related to tracheal collapse [6]. Other studies report that the bronchi most frequently affected by CBM are in the left lung, and especially its cranial lobe [5, 7, 8], consistently with our results.

The CBM diagnostic criterion we used (height to width ratio >2 of the lobar bronchi) is based on the study by Stadler et al. [12], and our CT images were obtained from dogs breathing under sedation. However bronchial collapsibility values, in terms of cross-sectional area, above 50% are found in healthy dogs, and are significantly greater during forced expiration than at the end of expiration [20, 21]. More rigorous diagnostic criteria based on more detailed data are thus needed.

Hypoxemia arises from a variety of mechanisms, such as V/Q mismatch, right-to-left shunt, diffusion impairment, hypoventilation, and low inspired PO_2_. We hypothesize that V/Q mismatch due to airway limitation in CBM is one of the causes of hypoxemia. Indeed, 9 out of the 11 dogs showed hypoxemia, with 6 mild and 3 severe cases. Abnormal lung patterns of one or both cranial lung lobes were observed in 6 dogs with mild hypoxemia. In addition, 3 dogs with moderate or severe hypoxemia had cardiogenic pulmonary edema due to severe mitral regurgitation, aspiration pneumonia, and suspected interstitial pneumonia, suggesting that moderate to severe hypoxemia occurred not only due to CBM, but also to lung lesions, since lung parenchymal disease is usually complicated by airway collapse [22, 23]. Normoxemia was observed in 2 dogs, which also presented with abnormal lung patterns. Notwithstanding their normoxemia, these 2 dogs may be affected by pulmonary dysfunction, as suggested by the elevated A-aDO_2_.

Normocapnia was found in 7 out of 11 dogs. Airway collapse involved only part of the bronchus, and might not have affected ventilation. Hypercapnia is often caused by brachycephalic syndrome. Blood gases analysis revealed that brachycephalic dogs have higher PaCO_2_ than do meso- or dolicocephalic dogs, and the dogs with PaCO_2_ > 35 mmHg were older (58 ± 16 months) [11]. The median age of dogs with brachycephalic syndrome in this study was 11 years, older than the dogs in the previous study [11]. One dog had hypercapnia, which could be attributed to brachycephalic syndrome in our studies. Hypocapnia in 2 of the dogs resulted from lower pH (7.297 and 7.382) and lower bicarbonate concentrations (11.4 and 16.9 mmol/L). Hypocapnia can be induced by agitation, fever, pain, lung disease, or compensation of metabolic acidosis, and the dogs examined in our study did not present with such factors.

A-aDO_2_ indicates the condition of the alveolocapillary membrane and the effectiveness of gas exchange, and thus is not affected by ventilation [24]. In humans, A-aDO_2_ increases with age due to the age-induced decrease of PaO_2_ levels due to the rise in V/Q mismatch [25]. In a previous study, geriatric dogs (mean age: 12.3 years) showed higher A-aDO_2_ than young dogs, but still < 21 mmHg [26]. The median age of the dogs in our study was 11 years, so we defined elevated A-aDO_2_ as >21 mmHg. As a result, all dogs had elevated A-aDO_2_ and abnormal lung patterns.

The abnormal lung patterns found in this study, such as parenchymal band and atelectasis, depended on the CBM site. Parenchymal band was the most commonly found lung pattern. It consists in a linear opacity, usually 1–3 mm thick, extending to the visceral pleura. It shows fibrosis, atelectasis and distortion of lung architecture [27, 28]. Moreover, previous studies reported brachycephalic dogs with collapsed cranial lobe bronchus. In this study, many brachycephalic dogs were enrolled, and the cranial lobe bronchi were the most affected, consistently with previous studies. Lorenzi et al. argue that CBM is associated with lung lobe torsion in brachycephalic dogs [8]. Our study reveals that atelectasis depends on the region of CBM, supporting the hypothesis that the lobe affected by atelectasis increases its mobility, resulting in lung lobe torsion. One dog had a mosaic pattern during the expiratory phase, during which, in normal cases, the whole lung shows increased attenuation, while patients with small airway disease shown heterogeneous attenuation due to air trapping [29]. In this case, the whole lung except the right cranial and caudal lobes showed low attenuation with pulmonary emphysema (Fig 2). Air trapping resulted in obstruction during the expiratory phase due to the collapsed bronchus, and caused pulmonary emphysema. Therefore the elevated A-aDO_2_ in CBM suggests that all dogs in this study had some pulmonary dysfunction due to air trapping.

A limitation of this study is the lack of comparison of dynamic CT with bronchoscopy and fluoroscopy. However, dynamic CT is noninvasive, can detect other lung abnormalities within a wide window, and does not expose the dog to radiation or drugs, and thus can be considered the ideal method. Another limitation is that no histopathology was obtained, so that it is not possible to compare CT and histopathology findings: However histopathological examination is difficult in clinical cases. Finally, another limitation of this study is the small sample size, so further studies are needed to confirm our results.

### Conclusion

Our study shows that CBM in dogs contributes to pulmonary dysfunction through oxygenation but not ventilation. Blood gas analysis, and especially the evaluation of A-aDO_2_, can indicate pulmonary dysfunction in dogs with CBM. However, the cause of decreased pulmonary function needs to be examined in detail.

## Supporting information

S1 Table Profiles of eleven dogs with bronchomalacia. (XLSX)

S2 Table Blood gas data sets of eleven dogs with bronchomalacia. (XLSX)
